# A method for creating interactive content for the iPod, and its potential use as a learning tool: Technical Advances

**DOI:** 10.1186/1472-6920-7-32

**Published:** 2007-09-22

**Authors:** Edward J Palmer, Peter G Devitt

**Affiliations:** 1 Centre for Learning and Professional Development, University of Adelaide, Adelaide, Australia; 2 Disclipline of Surgery, University of Adelaide, Adelaide, Australia

## Abstract

**Background:**

Podcasting is currently a popular means of delivery of information with a large number of podcasts specifically tailored for educational purposes. It can be argued that the passive nature of this teaching methodology limits the educational benefit that can be derived from podcasts. This paper describes the development and construction of interactive material for the iPod, and a survey of student attitudes towards this type of learning material.

**Methods:**

The development of interactive material for an iPod is described in detail. This material was developed and demonstrated to 50 medical students. These students completed a paper-based survey on the potential uses of this technology, before and after a 20 minute presentation in class of an interactive case-study on an iPod.

**Results:**

A technical description of how to develop interactive content for the iPod was created. The results of the student survey indicate a favourable shift in student attitudes after viewing the interactive case. Despite only 15% of the students owning an iPod, 57% of the students were positive about having access to interactive iPod content and 59% believed they would use it whilst travelling. The percentage of students who felt podcasting was a useful means of learning increased from 9% to 41%.

**Conclusion:**

The development of interactive content for the iPod is feasible. There are indications that students view interactive iPod cases as having value as an additional learning resource.

## Background

The arrival of new technology, particularly in the entertainment field, is often followed by efforts to leverage this technology for educational purposes. The introduction of records, radio, audio tape, video tape, CDs and laserdiscs have all been followed by educationalists taking up each of these tools and using them for their students' benefit [1-6]. The introduction of mp3 players, such as Apple's iPod, is proving to be no exception. Educational podcasts, which are audio recordings of items such as lectures, interviews and book readings are increasing in number [7,8], and currently make up about 7% of the total number of podcasts available for downloading. The sheer volume of material produced for the educational arena continues apace.

In the majority of cases, a podcast is essentially a passive learning experience focused on an audio facility alone. The video equivalent, the vodcast (video podcast), uses additional media and is therefore likely to be more versatile and of interest to the student but is still a passive learning tool. The main benefits of this type of learning material are the ease with which it can be created [9], downloaded and used [10], and its portable nature. This could provide significant benefits for students and clinicians separated from their peers by distance [10]. It is possible to create medical content in a short space of time and have it downloaded by thousands of people shortly afterward [11] and equally possible that students on public transport might choose to listen to, or watch educational material, in addition to watching videos or listening to their favourite music.

It has been suggested that non-interactive audio and video are less effective educational tools than computer aided learning [12], where the latter has additional scope for interactivity. If this interactivity really provides an educational benefit, it would make sense to try and give students the opportunity to use interactive material on their mp3 player. Some material is available for the iPod that provides a degree of interaction through links to an external web site [13], but it might be considered more beneficial to provide a portable, self-contained, interactive experience. The focus on the development of content for the iPod is important, as this is the mp3 player with the greatest market penetration, in a market dominated by young adults particularly in the demographic containing students [14-16].

We have developed an interactive process to be used on the iPod, which mirrors that of the computer-based Medici program [17], an interactive medical education program developed at the University of Adelaide. Medici is a dynamic, case-based, educational package consisting of problem solving exercises in clinical medicine [18]. The material in Medici spans the basic sciences from anatomy through to the clinical disciplines of radiology, ophthalmology, dermatology and neurosurgery, with the main focus on general medical and surgical topics. A typical problem solving exercise begins with an introductory screen, which displays the case title and details of the case author. Each section of the case consists of some information relating to the current status of a patient, a question about the management of that patient and a series of choices, which can be selected. Navigation from one part of the case to the next is handled by the click of the mouse. At every step of the way through a case, feedback is given about the choices made. Also provided is feedback about choices, which should have been made and were not. At any point in a case there may be images. Digital video is also available in Medici and is often used to illustrate medical procedures. When a Medici case is completed, a score is calculated and a critique is displayed. The critique summarises the material covered in the case, the learning issues and often includes useful references for further reading.

This paper describes, in detail, the methods to be used to construct interactive learning material for the mp3 player market, specifically the iPod, utilising the Medici methodology. As the authors wished to include images and video in their interactive material, the latest iPods (30 GB and second generation nano versions) [19,20] with high quality video screens have been used. The same methodology will work on many of the earlier generations of iPods with screens, but developers will have to be satisfied with displaying text only. Additionally, some insight into the way students are likely to react to this type of educational innovation is provided.

## Methods

### Technical specifications for creating interactive content

**Figure 1:**
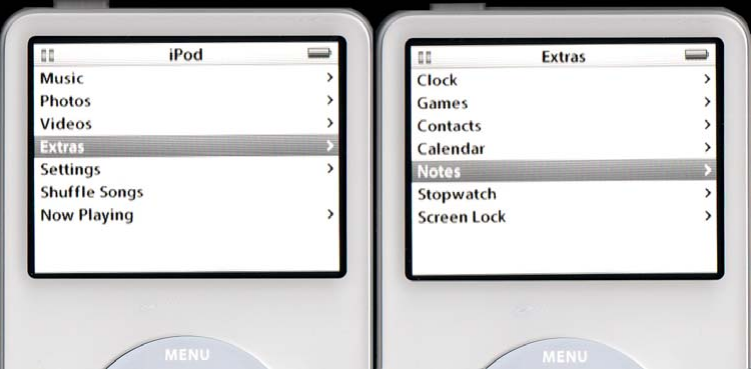
The Notes function on the iPod.

It is possible to enable the iPod to produce self-contained, interactive learning scenarios, supported by images, but there are some features in the Medici style of case based learning, which cannot be easily transferred into the iPod. These include interactive scoring and the ability of students to select multiple choices or enter free text. All other features can be translated onto the iPod. The little-used Notes function (figure 1) on the iPod is the key to providing interactive learning material. Within the Notes folder, documents supporting the html coding system, the code behind the world-wide web, can be run. This is used in train schedules [21], and games [22] that are run on the iPod and this function forms the base for interactive learning on the device.

**Figure 2:**
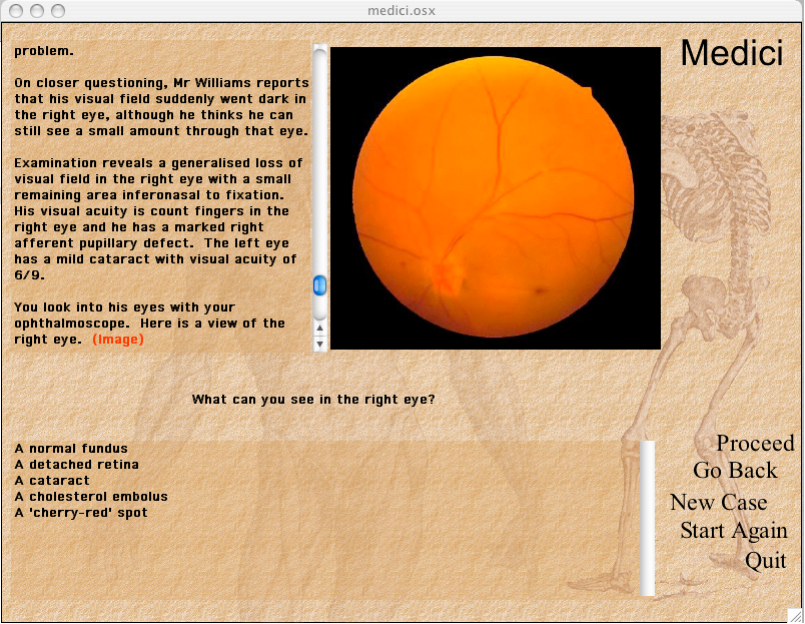
An ophthalmology case displayed in Medici.

Figure 2 shows part of the first page of an ophthalmology case in Medici. The image is an essential part of the case, and in this case the student is expected to select one choice from five options to answer the question posed. The question is based on data provided in the text and the image.

To run interactive content on the iPod, it is first necessary to render it into html format. To render educational content similar to that shown in Figure 2 into html code that the iPod can play, a series of steps were developed. The example in the results section illustrates the steps necessary to do this.

### Survey of student attitudes

To obtain an indication of how students might receive this type of learning material, fifty 5^th ^year medical students, in their second year of clinical study were surveyed regarding their views on using an mp3 player (and an iPod, in particular) as a learning resource. This survey was followed by a 20 minute lecture using a case study run as an iPod interactive case. The students were then surveyed again to see if their attitudes had changed. The students were asked about their use, knowledge and ownership of mp3 players in general and iPods in particular. The questions in this survey were 5 point Likert style. The choices available to students ranged from strongly agree (1) to strongly disagree (5). The 50 students were from a cohort of 130 students but comprised the entire class present at a lecture given prior to the iPod demonstration. The attendance figures for this class were similar to those of other lectures given in the teaching program and do not necessarily indicate a biased sample. Many of the students are attached to remote teaching hospitals and choose to remain in that location rather than attend a series of lectures.

The previous year, a similar survey had been performed amongst 39 5^th ^year students and the data is presented to determine any changes in attitude from one year to the next.

## Results

### Technical specifications for creating interactive content

Any plain text editor will suffice to develop interactive content for the iPod, as long as the user enters the html coding correctly. It is important to note that only a restricted subset of html code is supported. For example the bold tag <b> is not supported. The files created are transferred onto the iPod when it is mounted as a disk, by dropping them into the Notes folder or a subfolder.

#### 1. Develop content for the case

When developing graphical content for any educational system, care must be taken regarding copyright and patient consent. Case authors should not use any images from sources such as textbooks or web sites without permission and should not use images taken of patients without their permission. In our experience, patients are usually happy to consent to have clinical pictures taken, as well as images of test results, including radiological investigations, in order to help educate students.

##### a. Images via Adobe Photoshop or equivalent

Still images can be taken on any digital camera or from any other source, if permission is granted. Images will usually need to be edited. The authors use Adobe Photoshop [23], but there are many shareware and freeware packages, which can carry out simple editing functions. Often, digital cameras will come supplied with appropriate software. In general, any images, which could divulge a patient's identity, should have any identifiers removed (for example patient names on x-rays). Images appropriate for the iPod should have a minimum size of 320 × 240 pixels, which is the highest screen resolution of any iPod, and a resolution of 72 dpi. Unless there are plans to connect the iPod to a higher resolution screen, such as a television, there is no need to exceed these specifications. Images should be saved in JPEG Baseline format at a quality sufficient to display the required medical details.

##### b. Video. Edited by iMovie, Adobe Premiere or equivalent

Video can be filmed on any low to mid-end video recorder. A digital recorder, with a direct computer interface (firewire or USB2) will facilitate fast transfer of video to a computer and a cheap software package such as iMovie [24] on a Mac or Dazzle in Windows [25] will provide sufficient ability to edit the content. Expensive software packages such as Adobe Premiere [26] or Final Cut [27] can be used, but are not necessary. The video will need to be encoded to mp4 or H.264 standard with a size of 320 × 240. There is a setting in Quicktime Pro [28] specifically for this purpose (Export to iPod). Unless there are plans to connect the iPod to a higher resolution screen, such as a television, there is no need to exceed these specifications. The audio component of the video should be encoded using Advanced Audio Coding (AAC) (44.1 or 48 kHz, up to 160 kbit/s, stereo) [29]. Care should be taken to ensure that videos are as small as possible as students will need to download this material. A 2 MB file could take a student with dial up access 11 minutes to download.

##### c. Audio record using phone, dictaphone, PDA

Audio can be recorded as part of a video and be extracted from the video/audio package using software such as Quicktime Pro [28]. Dedicated audio recorders will also provide good quality mp3 files. Authors should consider investing in quality microphones if they plan to provide a large amount of audio content. The audio should be encoded using AAC (44.1 or 48 kHz, up to 320 kbit/s, stereo) [29].

#### 2. Provide a title for the case

The titles are displayed in alphabetical order in the iPod, so careful naming of the cases is required. A typical example of how to code this is shown below

• <title>Sudden loss of vision in an elderly man</title>

**Figure 3:**
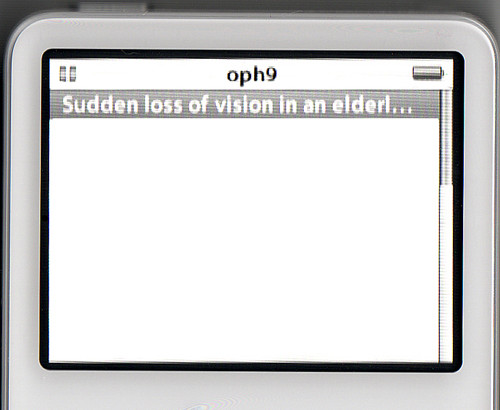
Case title displayed within the Notes folder.

The case will then appear on the iPod within the Notes folder as illustrated in Figure 3. Cases can be provided in a nested structure of folders. In this case, the case was located inside a folder called oph9. This is indicated at the top of the iPod screen. The case is selected using the scroll wheel and the centre button on the iPod.

#### 3. Provide information (text and/or image) for the case

There are no specific requirements when adding content. The iPod will ensure that any text wraps and is scrollable. The content can be pasted directly after the title. The <p> tags can be used to enforce line breaks.

• <title> Sudden loss of vision in an elderly man</title><p><p>Mr Williams is a 67 year old caucasian who presents with a four hour history of sudden visual loss. He was...

**Figure 4:**
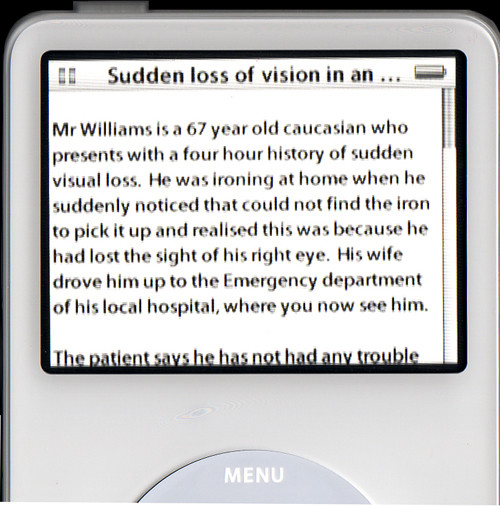
Case information displayed on the iPod.

This content will need to be saved as a plain text file within the case folder. Once a case has been started, this content will be placed on the screen as shown in figure 4.

#### 4. Link images into the case

**Figure 5:**
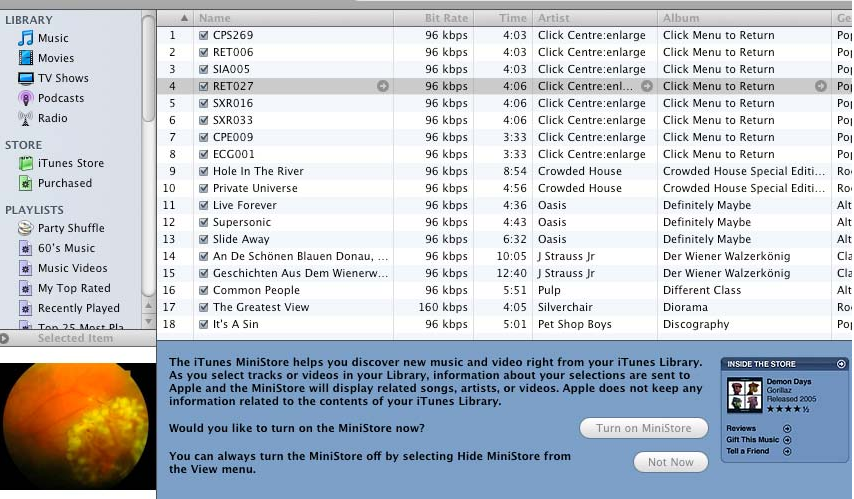
Images allocated to a case using iTunes.

Images can be referenced directly on the iPod using <a href="\enclosing folder(s)\picture name">(Image)</a>. To use this coding, the images need to be in the Notes folder and with the .jpg extension. This procedure works for the 30 GB iPod, but not for the iPod nano. For both types of iPods, it is possible to assign an image to a music track as if it were cover art. Figure 5 illustrates this process. The authors created new music tracks by copying existing music tracks then deleting the sound track. This leaves a 0 second music track, but allows for the addition of an image and provides the opportunity to reference the image from learning cases. The instructions on the screen "Click Centre:Enlarge" and "Click Menu to Return" are made available to users by manipulating the Artist and Album field in the iTunes program (Figure 5). Selecting this option also allows the developer to provide explanatory audio to accompany the image, which may be of benefit in some cases.

**Figure 6:**
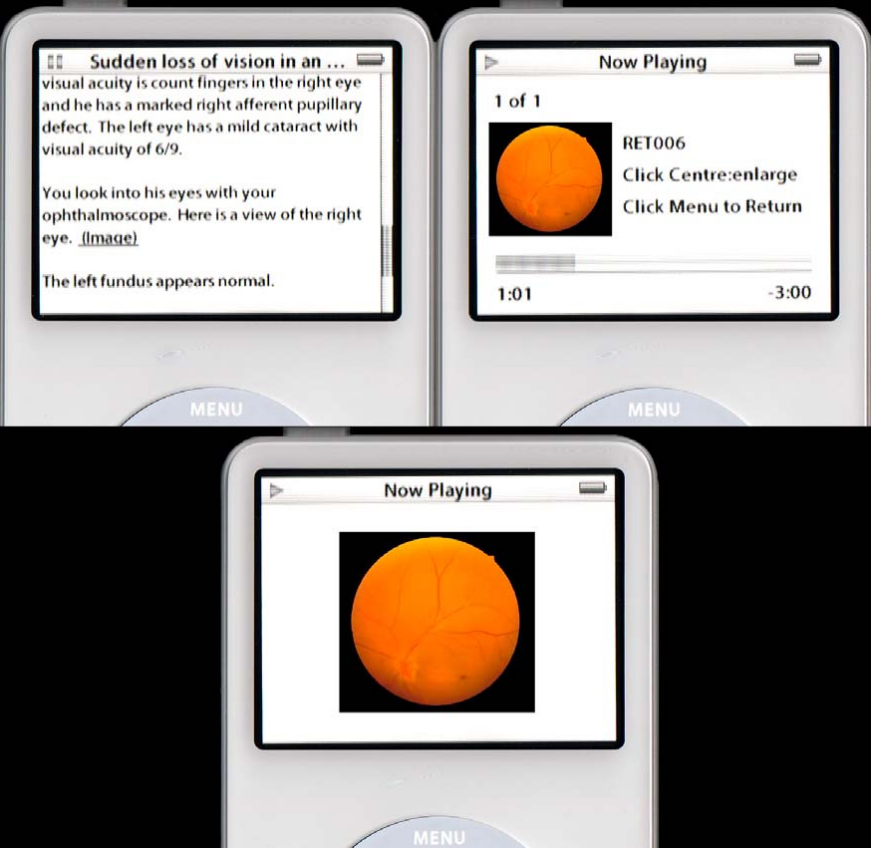
Images displayed within a case on the iPod.

Refer to the image in the text by using the <a href> tag and using some specific code designed for the iPod. The code directly calls up the music track RET006, which has the cover art describing the patient's eye. Figure 6 shows the result of this process on an iPod.

The image is a view of the right eye. <a href="ipod:music?song=RET006"> (Image)</a>

Video can be referenced in the same way using <a href="video=Video Name">(Video)</a> [30]. In this case there is no need to manipulate cover art nor have the videos located in the Notes folder. The videos are accessed directly.

#### 5. Add the question and the choices that can be made

**Figure 7:**
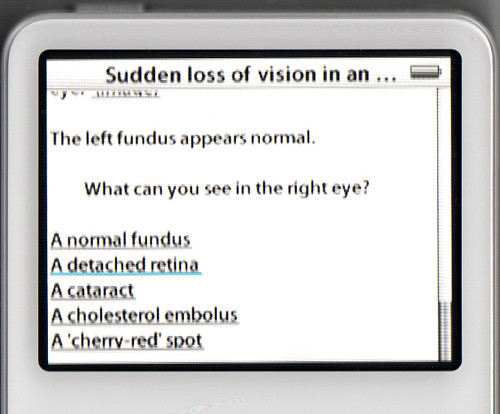
Selectable choices in an iPod case.

Each choice is a link to another web page, where the feedback for that choice can be supplied to the student. The coding for the choices is <a href="web page name">Name of choice</a> with an example shown below. The result of this is shown as Figure 7

The left fundus appears normal.<p><p>What can you see in the right eye?<p><p><a href="OPH9_1_1.html">A normal fundus</a><p><a href="OPH9_1_2.html">A detached retina</a><p><a href="OPH9_1_3.html">A cataract</a><p><a href="OPH9_1_4.html">A cholesterol embolus</a><p><a href="OPH9_1_5.html">A 'cherry-red' spot</a>

Note that it is important to maintain a consistent naming convention for the web pages designed for the iPod. In this case, the uppercase OPH9 indicates a short name for the case as allocated by the authors. The first digit "_1" indicates the first page of the case and the second digit indicates the choice number. The coding for the ahref tag is <a href="web page name">Name of choice</a>

#### 6. Provide feedback to the choices

Design the feedback for each choice as a separate web page and link each of these to the next step in the case. Each of the feedback pages requires a title and may have images associated with it.<title>OPH9_1_2</title>A detached retina. Score:0<p><p>Incorrect. Here is an example of a detached retina. <a href="ipod:music?song=RET027"> (Image)</a> Laser burns can be seen around the detached area.<p><p><a href="initialise.html">Go Back</a> <a href="OPH9_2.html">Continue Case</a>

#### 7. Deliver content to students

Content can be delivered to students in two ways. The content can be collected together, compressed and made available for downloading. Students will then need to load images and text files into their Notes folder and drop videos into their Movies library within iTunes [31]. Alternatively students can download the material as a series of podcasts. Setting up a podcast server will require an IT proficient person, but is a relatively straightforward task, described in detail in other forums [32].

To create a three-screen case such as this using a text editor would take approximately one hour. It would take the students a few minutes to download the case and additional media, assuming videos are used sparingly. The authors have created an option in the Medici program to save the cases directly to iPod format, so existing material can be converted very rapidly and be developed in the Medici authoring system.

### Survey of student attitudes

Fifty six percent of the students surveyed in 2007 (n = 50) had mp3 players (Table 1) but only 18% of the whole group owned an iPod with a video screen, which would allow them to run interactive material. Twenty percent of students downloaded podcasts. Even though 20% of students claimed to have downloaded podcasts, twice that number (41%) felt that podcasts would be a useful way of learning material. 47% had no opinion or believed this item was not applicable. Almost half the students (47%) felt that they would like access to interactive learning material to download to an iPod and 55% believed they would use an iPod to learn whilst travelling.

**Table 1:** Comparison of survey results for 5^th ^year students 2006/2007

	2006	2007
Total number of students surveyed (100% response rate)	39	50
Do you own an mp3 player?	Yes: 34%	Yes: 56%
Do you own an iPod?	Yes: 15% (any kind)	Yes: 18% (with video screen)
Do you download podcasts?	Yes: 18%	Yes: 22%
Podcasts are a good way to learn material	9% agree or strongly agree	41% agree or strongly agree
Would use iPod to study whilst travelling	n/a	55% agree or strongly agree
CD preferred method to aid learning (selection from CD, web, iPod)	68%	67%

When compared with the previous year's cohort of students (n = 39), some differences were apparent. (Table 1) Only 34% of the previous cohort had mp3 players (15% iPods), 18% had downloaded podcasts, 9% thought it was a good educational methodology and 15% thought it would be good to have educational material to learn whilst travelling. Sixty eight percent felt that the CD was the best method to deliver interactive material.

Table 2 shows the pre to post changes in the questions, which were common to both surveys. There was a change of 10% in the students who agreed or strongly agreed with the question asking if students would like to access interactive iPod material.

**Table 2:** Changes in attitudes after iPod lecture

	Pre Survey Agree+Strongly Agree (%)	Post Survey Agree+Strongly Agree (%)
I would like to have access to interactive learning material that I could download to an iPod	47	57
I would use an iPod with educational material on it to study whilst travelling (on bus, train, car etc).	55	59

Students were also asked to rank four different methods they would like to use to supplement their learning. The rankings were from 1 to 4 (1 highest) with no number repeated. Table 3 shows the results of this ranking process with the average number of the rankings shown. The highest ranked learning tool pre-lecture was interactive content on CD, followed by web sites, text books and iPods. Post lecture, the order remained the same but the perception of the value of interactive content on an iPod changed significantly as tested by the Kruskal-Wallis test of ranks.

**Table 3:** Changes in student rankings of learning methods. Methods rated from 1 to 4 where 1 is the highest rating

Ranking of educational learning methods	Pre Lecture	Post Lecture	Significance
Text Books	2.6	2.8	ns
iPod	3.5	3.0	0.03
CD	1.8	1.9	ns
Web site	2.2	2.4	ns

Students were asked to explain their rankings and any reason behind any changes. Thirty five percent of students ranked the iPod poorly, but only for the reason that they did not have an iPod. One fifth of students remarked that accessibility was their main reason for their ranking, although students were unable to agree on what accessible meant. For some students, CDs were more accessible as they could be taken anywhere and did not rely on internet access being available. For others, web sites were more accessible, because the students did not need to carry anything with them. Others preferred text books because they were not dependent on the internet or technology in general and they preferred to read off of paper and write notes as they read. The costs of items were also regarded as relevant, with students finding internet access, download costs and text books too expensive. In the pre lecture survey only one student felt that the iPod was the best way to deliver learning material. After the lecture, five students rated the iPod best, with the comments suggesting that the portability was the main attraction. The interactivity and the ability to receive feedback were noted as positive factors. Students also reported that they would seriously consider getting an iPod to aid their studies.

## Discussion

The idea of using an iPod as a learning tool resonated strongly with the students after seeing how it worked, although as many students pointed out, the lack of an iPod rendered the question not applicable to their situation.

Educators have developed content for the iPod which displays some interactive characteristics. Some podcasts have been constructed to use in conjunction with web links to images and other media, but these are not truly interactive, nor are they portable as they require an internet connection [13].

The ability to create interactive material is an important step forward for portable learning, but it is equally important that there is an audience for it. Although the survey relates to a small sample over two years, the indications from the results are that students are currently not sufficiently well equipped to use interactive learning material on an iPod. Students do see the benefits in using an iPod for podcasts, and more interactive learning. Interactivity, such as that gained from some CD based educational programs was regarded by the students as a valuable commodity and in the post lecture discussion, there was an overwhelming push for more medical material to be made available in this mode. The comparison with the previous year's students indicates that attitudes towards the educational possibilities of mp3 players appear to change as they are adopted to a greater degree, but the older methods still dominate student thinking.

iPods are not the only mp3 players. The iRiver Clix [33], sanDisk's Sansa [34] and Microsoft's Zune [35] are serious competitors for this market. Not all mp3 players have the capability to run html, and the methodology described in this paper may not be suitable for other mp3 players, but there are a number of PDAs and mobile phones which can run this content and they can do so live. This latter point is of great importance. The iPod is not an internet active device, so any medical content needs to be downloaded, and may therefore become out of date without the student's awareness. A "live" system, where content can be controlled and regularly updated has a great deal of appeal from a quality control perspective. The ability of some mp3 players and other devices to run Flash [36] applications greatly enhances the opportunities for meaningful interaction on a portable device and should be explored in greater detail. The release of newer technologies, such as Apple's iPhone [37] will also have a large impact on this type of interactive content.

## Conclusion

We have described in detail a method to allow teachers to create interactive learning material on the iPod. It appears that although the technology for truly portable learning is here, although in an as yet unrefined form, the technological capabilities of our students and their ability to purchase the technology may not quite be ready for a large investment in this area. There is also no evidence at this point in time to suggest that learning in this way is effective, The ability to retain knowledge and to apply concepts being taught is untested where students are no longer in a controlled learning environment such as the classroom or their home. This is clearly an important avenue for further research.

## Competing interests

The author(s) declare that they have no competing interests.

## Authors' contributions

EP was responsible for the technical design and survey analysis and involved in all drafts of the manuscript. PD was responsible for survey design and execution and course content and involved in all drafts of the manuscript. Both authors read and approved the final manuscript.

## Pre-publication history

The pre-publication history for this paper can be accessed here:


